# Upgrading Pyrolytic Residue from End-of-Life Tires to Efficient Heterogeneous Catalysts for the Conversion of Glycerol to Acetins

**DOI:** 10.3390/molecules28248137

**Published:** 2023-12-17

**Authors:** Anna Malaika, Jolanta Kowalska-Kuś, Klaudia Końska, Karolina Ptaszyńska, Aldona Jankowska, Agnieszka Held, Krzysztof Wróblewski, Mieczysław Kozłowski

**Affiliations:** 1 Faculty of Chemistry, Adam Mickiewicz University in Poznań, Uniwersytetu Poznańskiego 8, 61-614 Poznań, Poland; amalaika@amu.edu.pl (A.M.); k.konska@contec.tech (K.K.); karolina.ptaszynska@amu.edu.pl (K.P.); aljan@amu.edu.pl (A.J.); awaclaw@amu.edu.pl (A.H.); 2 Contec, al. Jerozolimskie 142A, 02-305 Warszawa, Poland; k.wroblewski@contec.tech

**Keywords:** waste tire recycling, thermal pyrolysis, recovered carbon black, glycerol acetylation, carbon functionalization

## Abstract

Recovered carbon blacks (rCBs) produced from end-of-life tires using pyrolysis were transformed into solid acid catalysts for the synthesis of acetins, i.e., products with a wide spectrum of practical applications. Tuning the chemical properties of the surface of samples and introducing specific functional groups on the rCBs were achieved through carbon functionalization with concentrated H_2_SO_4_. The initial and modified rCBs were thoroughly characterized using techniques such as elemental analysis, potentiometric back titration, thermogravimetric technique, scanning and transmission microscopy, X-ray photoelectron spectroscopy, etc. The catalytic activities of the samples were measured via batch mode glycerol acetylation performed at 110 °C and compared to the catalytic performance of the functionalized commercial carbon black. The modified rCBs were found to show a significant catalytic effect in the tested reaction, giving high glycerol conversions (above 95%) and satisfactory combined yields of diacetins and triacetin (~72%) within 4 h; this behavior was attributed to the presence of -SO_3_H moieties on the surface of functionalized rCBs. The reusability tests indicated that the modified samples were catalytically stable in subsequent acetylation runs. The obtained results evidenced the feasibility of using end-of-life tires for the production of effective acid catalysts for glycerol valorization processes.

## 1. Introduction

The disposal of waste tires has become a significant environmental concern, given their abundance and the challenges associated with their proper management. In the United States alone, it is estimated that over 300 million waste tires are generated annually, with approximately 1 billion generated globally [1,2]. The accumulation of tires in landfills poses several environmental risks, including the potential for groundwater contamination, fire hazards, and the creation of breeding grounds for pests. Efforts to address the issue of tire waste have focused on finding sustainable and efficient solutions for tire disposal and recycling. One of the most promising approaches is the valorization of waste tires through pyrolysis, which allows for the conversion of the rubber and other components into liquid and gaseous fractions as well as a carbon-rich solid known as recovered carbon black (rCB). 

Pyrolyzing end-of-life tires (ELTs) into rCBs offers several advantages in tackling tire waste. Firstly, it provides a sustainable and eco-friendly method for repurposing discarded tires. Instead of ending up in landfills or causing environmental harm, the tires are transformed into valuable carbon materials that can be used in numerous ways. This approach contributes to waste reduction and resource utilization. Secondly, rCBs are quite similar to industrial carbon blacks (CBs) and can replace them in several applications, making the existing technologies more profitable. Finally, the rCB production processes use waste tires instead of fossil fuels (as opposed to virgin CB production technologies), which significantly decreases the carbon footprint and air pollution compared to the traditional CB manufacturing process. 

A notable application of rCBs and their activated counterparts is in the field of catalysis. These carbon materials possess a high surface area, well-developed porosity, and a complex structure prone to chemical functionalizations, providing an excellent platform for catalytic reactions. They can be utilized as catalyst supports [3,4,5,6] or active catalysts on their own [7,8,9,10], enabling various chemical transformations. 

Modification of carbons through sulfonation is a widely studied approach in the field of carbon material research. This method involves the introduction of sulfonic functionalities (-SO_3_H) onto the surface of carbon, resulting in acidic sulfonated carbon solids. This modification process offers several advantages and enhances the properties and applications of the carbon materials. The presence of sulfonic acid groups provides sufficient acidic strength to effectively catalyze processes such as transesterification [11,12,13,14], esterification [15,16,17], hydrolysis [18,19,20], and etherification [21,22] among others.

The modification of carbons via sulfonation can be achieved through various methods, including the use of sulfuric acid [23,24,25], 4-benzendiazonium sulfonate (4-BDS) [26,27], and 1,4-butane sultone precursor as sources of sulfonic acid groups [28]. The choice of sulfonation method depends on the carbon type, the desired level of sulfonation, and the specific application requirements.

The utilization of solid acid catalysts in industrial processes has garnered significant attention in recent years and has become one of the most prominent research areas in chemistry. One notable industrial reaction that requires novel solid catalytic systems is the process of glycerol valorization. Glycerol is produced as a by-product during biodiesel synthesis, resulting in an excess supply of glycerol in the market [29]. This surplus has implications for the economic feasibility of biodiesel production technology. To address this issue and enhance the viability of biodiesel synthesis, various strategies have been explored to effectively utilize the excess glycerol [30,31,32].

Among the different options, the acetylation of glycerol with acetic acid has emerged as an intriguing approach. This reaction leads to the formation of glycerol acetates, commonly referred to as acetins, including monoacetin, diacetin, and triacetin (MA, DA, and TA, respectively) [33,34,35]. These acetins hold significant importance as value-added chemicals with diverse industrial applications. They find use in various industries, ranging from cosmetics to the production of biodegradable polymers [36,37]. Furthermore, acetins (especially DA and TA and their mixtures) have potential applications in the fuel market, particularly as promising blending compounds [38,39]. Therefore, the acetylation of glycerol with acetic acid presents a valuable opportunity to transform the surplus glycerol into commercially significant products. 

The valorization of glycerol with acetic acid often relies on heterogeneous catalysts, such as heteropolyacids supported on various substrates [40,41], ion-exchange resins [42,43], sulfated zirconia catalysts [44], or silica-based systems [45,46]. Although there is also a significant number of papers on the utilization of carbon materials in this specific reaction [25,35,47,48,49], to the best of our knowledge, this is the first report on the application of tire-derived carbons for glycerol acetylation. Considering the significant industrial importance of glycerol acetylation and the need for the utilization of waste tires, this study focused on the development of new highly efficient carbon catalysts that can be easily prepared from problematic waste sources.

## 2. Results and Discussion

### 2.1. Characterization of the Samples

**Figure 1:**
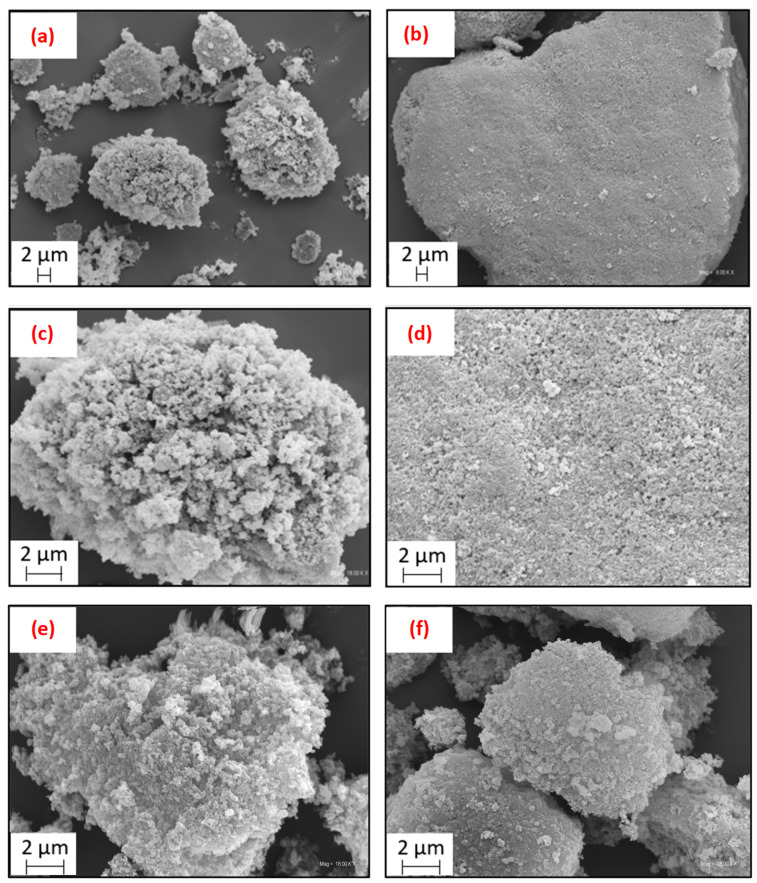
Scanning electron microscope (SEM) images of rCB_1 (**a**,**c**), rCB_2 (**b**,**d**), rCB_1-SA (**e**), and rCB_2-SA (**f**).

The morphology of the pristine rCBs (i.e., rCB_1 obtained through periodic pyrolysis and rCB_2 being a product of so-called Molten technology) is displayed in Figure 1. For the sake of comparison, SEM images of a commercial carbon black (CB) are also shown (Figure S1 in Supplementary Material). 

In Figure S1a,b, it can be observed that a commercial sample displayed characteristic spherical primary particles, typical of carbon blacks, fused together to form aggregates and agglomerates of irregular shapes and considerable sizes. Additionally, some free spaces and channels were visible in the material structure, giving the sample morphology a resemblance to a coral reef.

At first glance, the morphological features of rCB_1 and rCB_2 differed significantly from those of the commercial CB. Figure 1a, representing rCB_1, and Figure 1b, representing rCB_2, show that the obtained tire-derived samples consisted of compact particles of several micrometers and oval or irregular shapes.

The larger particle sizes were observed in the case of rCB_2 produced through continuous pyrolysis. This sample also presented a more homogeneous and “dense” particle surface. On the other hand, in the case of rCB_1 obtained in a batch process, the particle structure resembled that of a cauliflower, with a surface roughness morphology.

Higher-magnification SEM images presented in Figure 1c (representing rCB_1) and Figure 1d (representing rCB_2) exhibited that the produced recovered carbon blacks showed some structural similarity to commercial CB, as tiny spherical primary particles were visible in both rCB_1 and rCB_2 samples. Interestingly, some brighter spots, attributed to the presence of inorganic compounds [50], were also observed in the images. The primary particles formed a three-dimensional network of aggregates, with some inner spaces between the formed structures in the case of rCB_1 produced in batch pyrolysis (Figure 1c). On the other hand, the continuous Molten technology was observed to result in the formation of more aggregated particles and solid surfaces (Figure 1d). 

To verify the influence of the acidic treatment on the rCB morphology, scanning electron microscopy was employed to investigate rCB_1-SA and rCB_2-SA. The obtained SEM images of the modified carbons are presented in Figure 1d,f. As observed, the acidic treatment affected the morphology of the samples. For rCB_1 SA, the particle surface remained rough but appeared more compact compared to rCB_1. Furthermore, the micrograph of the modified rCB_1 carbon showed a lower number of bright spots (assigned to the presence of inorganic matter [50]) compared with the image of the raw material. This can suggest partial removal of inorganic matter from rCB_1 due to the sample’s acidic treatment. For rCB_2-SA, the changes in sample morphology primarily involved reduced particle sizes compared to the unmodified carbon.

**Figure 2:**
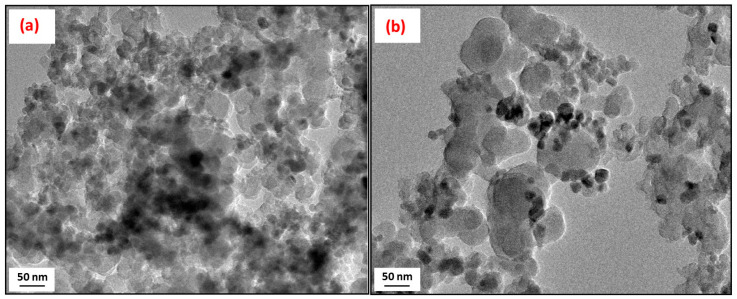
TEM images of recovered carbon black samples: (**a**) rCB_1 and (**b**) rCB_2.

The morphology of the pristine rCB samples was confirmed by the TEM micrographs obtained for these materials, presented in Figure 2. As can be observed, the produced rCBs were predominantly composed of nearly nano-sized spherical particles, each with a diameter of less than 100 nm, quite similar to those in commercial carbon blacks [51]. However, it is important to note that there was a broad and varied particle size distribution within the obtained rCB samples.

The results of the elemental analysis of the rCB and CB samples are presented in Table 1. As can be observed, both post-pyrolytic solids showed quite comparable compositions; however, they were different from that of commercial carbon black. The contents of carbon in rCBs were in the range of 75–80%, whereas a commercial CB sample contained 99.6% of C. Noticeably, rCBs also indicated quite a significant amount of S in their structures (as opposed to CB), i.e., 2.4–2.8%, which was due to the presence of sulfur in the tire feed material, coming from a vulcanization process. These S values were quite similar to those reported by other authors for the tired-derived rCBs [52,53]. Importantly, an essential parameter differing the obtained rCBs from the commercial carbon blacks was the quantity of ash; namely, rCB_1 and rCB_2 showed a considerable amount of ash (about 21–27%), whereas the commercial CB sample did not contain mineral matter. The presence of minerals and metals at the surface of the rCBs was also confirmed by the results of the microscopic studies using an EDX detector (Figure S2 in Supplementary Material). The obtained data revealed that the surface of rCBs contained several inorganic elements, notably Zn and S, in substantial quantities. Furthermore, some amounts of Ca, Na, Fe, and other elements were also found. It should be noted, however, that some differences were observed in the surface composition of rCB samples prepared according to different pyrolysis procedures. Importantly, as shown in Table 1, the total acidities measured for the commercial CB sample and rCB solids were close to 0 mmol H^+^/g, indicating that these materials did not contain acidic groups on their surfaces (both S- and O-containing). 

To induce surface acidity, the obtained rCBs as well as the commercial CB sample were functionalized with concentrated sulfuric acid. The results of elemental analysis, ash content determination, and total acidity measurements achieved for the initial and functionalized materials are depicted in Table 1. As can be seen, the sulfonation procedure resulted in obtaining samples showing higher sulfur contents compared to the pristine commercial and recovered CBs, i.e., in the range of 1.0–3.8%. Importantly, a direct effect of the applied functionalization on the samples’ surface chemistry was quite a significant increase in their total acidities (A_tot_; up to 0.76 mmol H^+^/g), which was most likely due to the introduction of surface sulfonic groups to rCBs and CB. It cannot be excluded, however, that acidic oxygen moieties were also formed during the functionalization of samples, as reported earlier for the sulfonation of various carbon-type materials [55,56,57]. In general, the obtained rCBs were more susceptible to modification with H_2_SO_4_, for which the A_tot_ values after the sulfonation were up to 0.76 mmol H^+^/g compared to 0.30 mmol H^+^/g for CB_SA. It is also worth noting that the use of concentrated sulfuric acid for sulfonation led to a significant decrease in the samples’ ash contents. Similar observations were also made by Cardona-Uribe et al. [58].

**Figure 3:**
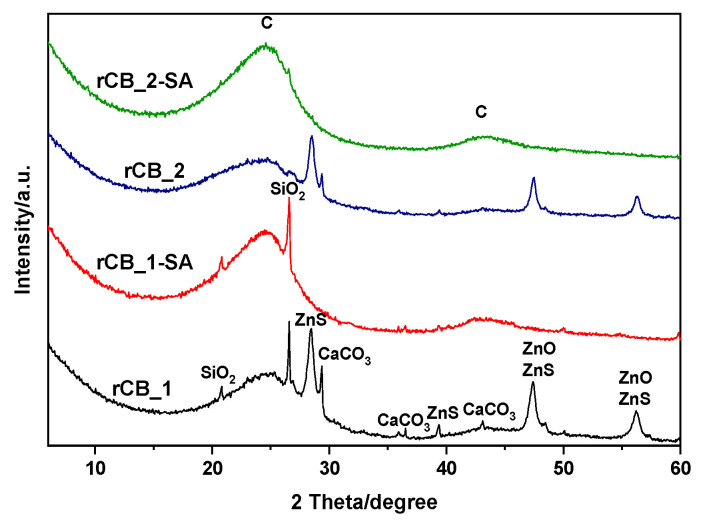
X-ray diffraction patterns of the tire-derived char: rCB_1 and rCB_2 and their sulfonated counterparts.

The crystallographic structure of the produced rCBs was examined using an X-ray diffraction technique. The XRD pattern of the pristine samples in Figure 3 revealed a broad signal at 2θ around 24° and a weak reflex at 2θ of about 43° (both indexed as ‘C’), typical for amorphous carbon materials [59]. Furthermore, the obtained diffractograms confirmed the presence of several impurities in the samples, which is also in line with the results of the ash determinations (see Table 1) and EDX analysis (see Figure S2 in Supplementary Material). For both pristine rCBs, diffractions at ca. 2θ = 28, 36, 48, and 56° were probably related to the presence of ZnS (indexed as ‘ZnS’) and ZnO (indexed as ‘ZnO’) [60,61]. On the other hand, the reflection at ca. 2θ = 29° observed in the pristine rCB_1 and rCB_2 samples indicated the presence of CaCO_3_. Additionally, the diffractogram of rCB_1 showed a strong reflection at 2θ equal to 26.6°, most likely associated with the presence of SiO_2_ (indexed as ‘SiO_2_’) [59,62,63,64]. All the compounds identified are added or formed in situ during tire manufacture. 

The diffractograms of the sulfonated rCBs differed from those of the pristine materials. Following the acid treatment with sulfuric acid, the peaks associated with the crystalline phases vanished (the exception was the rCB_1 sulfonated sample where the reflection at 2θ of ~26° was still present), confirming that the H_2_SO_4_ acid treatment can effectively eliminate the minerals and other impurities originally present in the samples. This finding is also in accordance with the results of the decreased ash contents noted for the functionalized rCB samples (see Table 1) and the SEM findings (Figure 1). 

**Figure 4:**
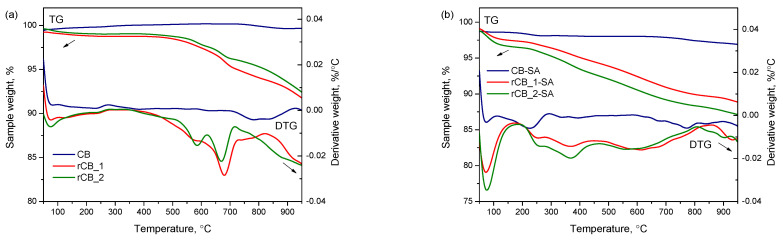
TG and DTG analysis: (**a**) the pristine commercial and recovered carbon blacks (i.e., CB, rCB_1, and rCB_2) and (**b**) CB and rCBs after the treatment with concentrated sulfuric acid.

Figure 4 presents the results of the thermogravimetric (TG) analysis performed for commercial and recovered carbon blacks, both pristine and after the acidic functionalization. As observed in Figure 4a, commercial CB exhibited minimal weight changes throughout the TG measurement. In contrast, the initial rCB samples displayed quite noticeable weight decreases which started to be significant at about 400 °C. Finally, the rCB samples lost approximately 7% of their initial weight. According to the DTG results in Figure 4a, the most remarkable weight losses were observed at about 580 °C and 680 °C, i.e., at temperatures higher than the temperatures of the sample preparation (see also Section 3.1). These signals were probably due to the decomposition of some less thermally stable inorganic additives as well as to the further carbonization of a rubber-derived carbonaceous solid.

The TG and DTG plots obtained for the functionalized samples are shown in Figure 4b. 

As presented, the DTG patterns of the sulfonated CB and rCB samples showed peaks at the temperature <100 °C, attributed to the release of water, and broad signals with the maxima at about 220, 250, and 380 °C, which can be ascribed to the presence of sulfonic and oxygen functionalities [57,65]. The appearance of the latter indicates the effective introduction of functional groups onto the surface of investigated samples. The smallest changes were observed for the commercial CB, suggesting the lowest degree of functionalization of CB-SA amongst the prepared samples, which is also in accordance with the results of the S and O contents in this material presented in Table 1. Interestingly, the signals originally present in the DTG patterns of the pristine rCBs in a temperature range of 400–700 °C were flatter in the DTG plots of the acid-treated materials. This can indicate a partial removal of mineral matter and impurities from rCBs and is also in line with the conclusions drawn from the ash determination, XRD, and SEM analyses.

To study the composition and the state of chemical species in the obtained rCBs, an X-ray photoelectron spectroscopy (XPS) analysis was performed. 

**Figure 5:**
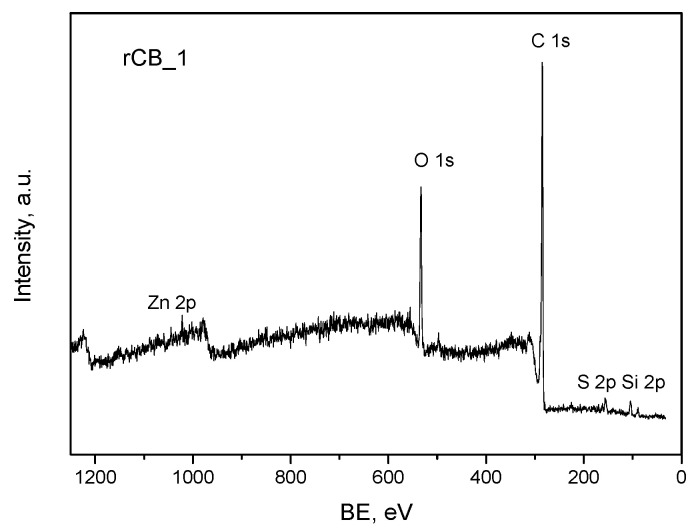
The XPS survey scan of rCB_1.

The XPS survey spectra of a selected rCB sample (pristine rCB_1) are depicted in Figure 5. Two clearly resolved C 1s and O 1s photoelectron peaks were observed. Furthermore, small photoelectron peaks originating from the presence of other atoms such as S, Si, and Zn were also reported. The presence of these elements on the surface of rCBs comes from an original recipe of tires and is also in accordance with the previous results (see the discussion on the ash, XRD, and SEM analyses).

**Figure 6:**
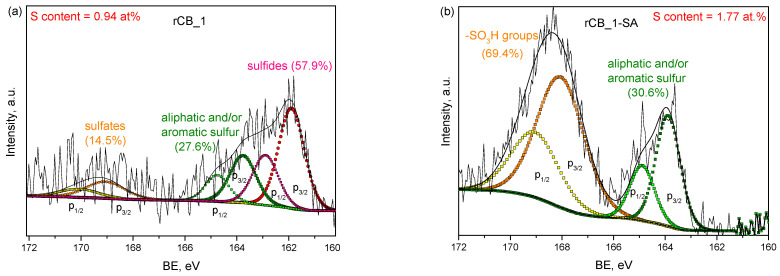
The high-resolution XPS S 2p spectra obtained for (**a**) rCB_1 and (**b**) rCB_1-SA.

The deconvoluted high-resolution XPS S 2p spectra of a selected sample before and after functionalization are shown in Figure 6. As observed, different sulfur species were present on the surface of the post-pyrolytic rCB_1 solid, namely the peaks at lower BEs (162–165 eV region) can be ascribed to the reduced forms of S, such as inorganic sulfides (BE~162 eV) and aliphatic or aromatic sulfur (BE~164–165 eV) [66,67]. The sample also contained some amounts of S in an oxidized state, probably in the form of sulfates, as indicated by the presence of a peak at a BE~169.5 eV [66]. Importantly, an intense signal at ca. 168 eV appeared in the S 2p spectrum of the functionalized rCB_1, suggesting the effective incorporation of -SO_3_H groups on the surface of this sample. This also agrees well with the increased A_tot_ reported for rCB_1-SA compared to that of rCB_1 (see Table 1). It is also clearly visible that some sulfur forms (the reduced ones; BE~162 eV) were leached from the prepared rCB_1 after its acidic treatment, which is consistent with the XRD data. The relative concentration of different sulfur species in the rCB_1 and rCB_1-SA is presented in Figure 6. The incorporation of -SO_3_H species onto the sample surface was also confirmed for rCB_2 (see Figure S3 in Supplementary Material). 

The high-resolution XPS C 1s spectra of the obtained rCB (raw and modified ones) are displayed in Figure S4 in the Supplementary Material. As presented, the virgin samples showed profiles typical for carbon materials, with a main signal at 284.6 eV assigned to sp^2^ and sp^3^ carbon [56]. Additionally, the deconvoluted spectra presented peaks at a BE of about 286.0 ± 0.2 eV, 287.4 ± 0.2 e, 289.5 ± 0.2 eV, and 290.5 ± 0.2 eV, typically ascribed to C-O, C=O, O-C=O, and pi-pi* transitions, respectively [68]. It seems, however, that in our case, the peaks in the 286.0–289.5 eV region corresponded at least partially to C-S, C=S, and CO_3_^2−^ moieties [69,70,71], as the virgin rCBs contained quite a lot of sulfur and inorganic matter in their structures (see also Table 1). Nevertheless, due to this complex composition of rCBs, the unequivocal assignment of the peaks is not straightforward. Further, as observed in Figure S4a,c), there were some differences in the spectra of rCB_1 and rCB_2, suggesting that the method of pyrolysis affected the sample surface composition, e.g., batch pyrolysis yielded a material with a higher contribution of functionalities assigned to the peaks at 286.0, 287.4, and 289.5 eV compared to rCB_2. Additionally, in both cases, the sulfonation of rCBs with concentrated sulfuric acid altered the rCB surface characteristics, removing some of the C-X moieties.

The results of the textural analysis of the pristine and modified samples are presented in Table 2. 

The relatively high BET surface area of 68 m^2^/g obtained for the commercial CB material was comparable to that of rCB_1 and rCB_2, which showed *S_BET_* values of 66 m^2^/g and 63 m^2^/g, respectively. These BET surface values obtained for the produced rCBs fall within the range of those previously reported for tire-derived solids [72,73] and resulted only from the presence of pores of higher sizes (as *V_micro_* in all cases was not detected). Furthermore, based on the analysis of the adsorption/desorption isotherms depicted in Figure S5 in the Supplementary Material, it can be inferred that rCB samples exhibited type IV behavior, characteristic of mesoporous adsorbents [74]. 

After the modification with sulfuric acid, the BET surface areas of the rCBs samples slightly increased, indicating a partial purification of the sample surface from unbound particles, referred to as ash [58]. This finding is also in line with the previous results (please refer to Table 1 and Figure 1, Figure 3 and Figure 4 for specific details). 

### 2.2. Catalytic Results

**Figure 7:**
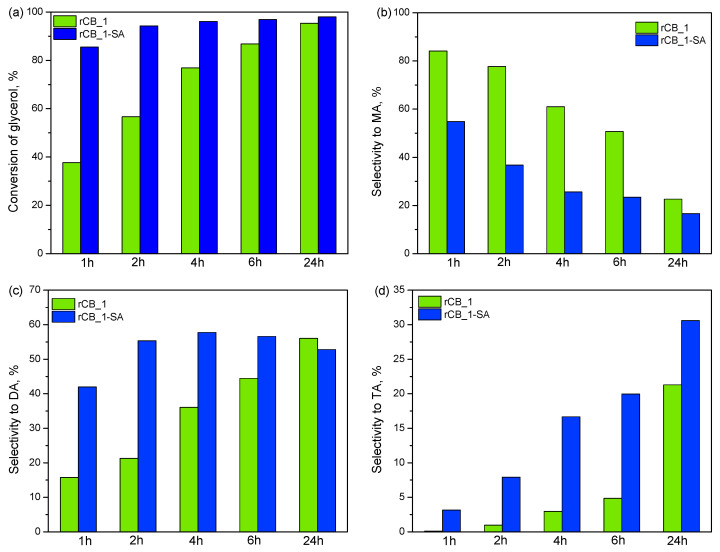
The influence of the modification of rCB_1 on its catalytic performance in glycerol acetylation versus time: (**a**) conversion of glycerol; (**b**) selectivity to monoacetins (MA); (**c**) selectivity to diacetins (DA); and (**d**) selectivity to triacetin (TA).

Figure 7 presents the catalytic results obtained in glycerol acetylation using a selected functionalized sample (rCB_1-SA) versus time (expressed as the conversion of glycerol and selectivity to individual acetins). To be sure that the observed catalytic effect is due to the sample modification and not the presence of mineral matter (see also the ash content for rCB_1-SA in Table 1), the pristine material (i.e., rCB_1) was also tested. 

**Figure 8:**
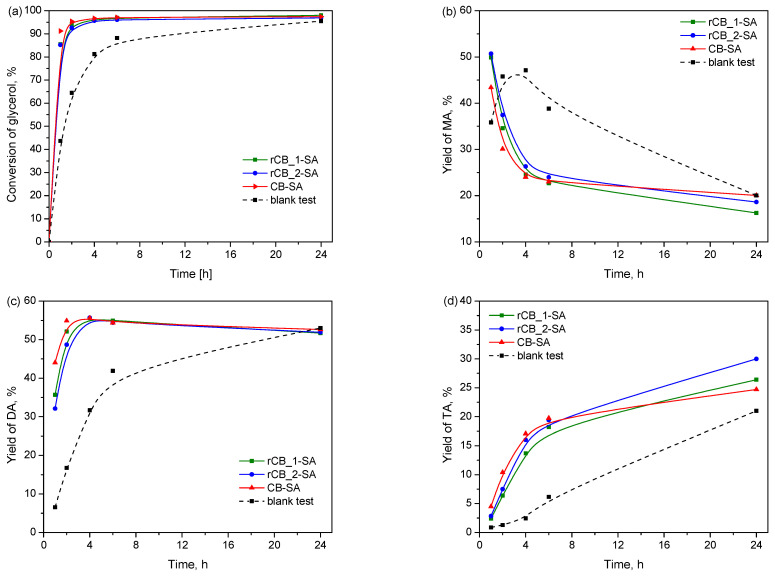
The catalytic activities of the sulfonated rCBs produced from waste tires in the process of glycerol acetylation in comparison to that of the modified commercial CB and the blank test expressed as (**a**) conversion of glycerol; (**b**) yield of monoacetins (MA); (**c**) yield of diacetins (DA); and (**d**) yield of triacetin (TA).

As can be seen in Figure 7a, the glycerol conversion obtained after 1 h of the reaction over unmodified rCB-1 was about 40%. This parameter increased with time; however, at each measuring point, the achieved results were comparable to those obtained for the blank (please see also Figure 8 and the relevant discussion). This simply means that the pristine rCB-1 (and the contaminants present in the sample) did not show any catalytic effect in the tested process. This finding agrees well with the negligible rCB_1 acidic properties (see Table 1) and the lack of acidic sulfonic groups in this material (see Figure 6a). rCB_1-SA showed an improved catalytic performance in the reaction compared with its unmodified counterpart. The observed enhancement in the sample activity was most likely related to the functionalization process and the introduction of acidic sites on the rCB-1 surface, as suggested by comparing A_tot_ values of rCB_1 and rCB_1-SA in Table 1 and XPS results in Figure 6. The same behavior was also reported for the rCB_2 pair of samples, i.e., raw (which was practically inactive in the reaction) and sulfuric-acid-treated (which showed an improved catalytic performance compared to the initial carbon).

Figure 7b–d depicts the distribution of different products obtained in glycerol acetylation over the pristine and modified rCB_1 versus time. As can be seen, mainly MA was initially formed when the untreated rCB_1 was applied. The use of rCB_1-SA as a catalyst resulted in considerable changes in the distribution of acetins, promoting the formation of mainly DA and TA products, i.e., the compounds of special interest. Furthermore, the selectivity to higher substituted esters generally increased with time at the expanse of MA, suggesting a subsequent transformation of MA to DA and TA. This observation is in accordance with the suggested mechanism of glycerol acetylation over carbon-type catalysts [75]. Finally, after 24 h, the combined selectivity to diacetins and triacetin obtained using rCB_1-SA was over 80%.

Figure 8 compares the catalytic results obtained in the reactions over the functionalized tire-derived carbons (i.e., rCB_1-SA and rCB_2-SA), modified commercial carbon black (i.e., CB-SA), and in the blank test (reaction without a catalyst). 

As observed, the reaction of glycerol with acetic acid is an autocatalytic process. This is due to the presence of a hydrogen atom in a CH_3_COOH compound. Thus, the blank test showed about 45% conversion of glycerol after 1 h, which increased with time to about 90% after 24 h. About a 38% yield of MA was obtained in the first 6 h of the reaction, while the yields of DA and TA obtained within this time were only about 40% and 7%, respectively. As can be seen, the functionalized recovered CBs worked efficiently in the process, giving significantly higher conversions of glycerol and yields of higher substituted acetins, i.e., DA and TA, compared with the reaction without a catalyst, in a short time. Thus, *Y_DA_* and *Y_TA_* obtained using modified rCB_1 and rCB_2 were about 53% and 18% after 6 h of the reaction. The extension of the reaction time resulted in only slight changes in the *Y_DA_*, whereas *Y_TA_* increased to about 25–32% after 24 h. Importantly, the catalytic performance of the produced catalysts was comparable to that exhibited by the modified commercial CB sample. This agreement aligns well with the similar sulfur contents introduced to the commercial CB (1.0%) and rCBs (increase by approximately 1.0% and 1.3%) after their modifications (see Table 1). 

**Figure 9:**
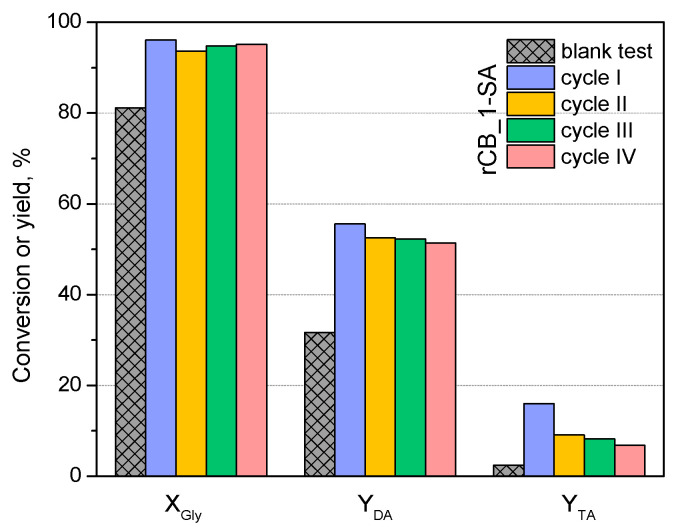
Reusability of a selected sample (rCB_1-SA) in four subsequent runs compared with the blank (*t* = 4 h); *X_Gly_*—conversion of glycerol, *Y_DA_*—yield of diacetins, *Y_TA_*—yield of triacetin.

To assess the catalytic stability of the obtained catalysts, a selected sample (rCB_1-SA) was tested in four subsequent reaction runs (time of reaction of 4 h), and the obtained results are presented in Figure 9. As can be observed in the graph, the conversion of glycerol practically did not alter when the catalyst was recycled, and in the fourth run, the *X_Gly_* measured after 4 h was still about 95%. Generally, the yields of the most valuable products, i.e., DA and TA, did not change considerably in the successive cycles, although some drop in the TA yield was observed after the first reaction run. Further changes in *Y_TA_* were not so significant, and the yield of triacetin obtained in the fourth reaction over rCB_1-SA was still higher than that produced in the blank. The slight drop in the catalyst activity was probably related to the partial leaching of the active sites of the reaction. This was suggested by the slight decreases in the S content and sample total acidity of rCB-1_SA after the fourth reaction cycle compared with the fresh rCB_1-SA (please compare the results in Table 1 and Table 3). Interestingly, the ash contents in the fresh and reused rCB_1-SA were comparable, suggesting that the mineral matter was not dissolved in the reaction medium. Therefore, an expensive purification step of the produced rCBs before their use in glycerol acetylation can be omitted.

Table 4 presents a comparison of the catalytic results obtained in glycerol esterification over tire-derived rCBs and other carbon-type catalysts. As can be observed, the glycerol conversions achieved using typical carbons described in the literature were very high and comparable (above 90%) in all cases. However, the samples exhibited varying activities towards the formation of DA and TA, and the conditions for obtaining satisfactory results differed. For example, de la Calle et al. [76] found that sulfonated hydrothermal carbon produced from glucose (SHTC) could give about a 60% selectivity to diacetins (DA) and a 30% selectivity to triacetin (TA) within 4 h. However, a large excess of acetic acid (Gly:AA molar ratio of 1:9) was necessary to achieve those results. Other types of carbons, such as C_glycerol obtained by partial carbonization with concentrated sulfuric acid or thermally reduced graphene modified with diazonium salt (TRGO_BDS), worked effectively at a more economically favorable Gly:AA molar ratio (1:6), giving selectivity to TA of about 20% within 2 h. On the other hand, the carbon produced from the Karanja seed shell at 400 °C (KJ-400) yielded only about 40% and 4% selectivities to DA and TA, respectively, within 4 h. In view of the above, the catalytic performance of the functionalized rCBs prepared in this research was very promising as the samples gave a significant combined selectivity to DA and TA (~75%) within a short time at an economically acceptable Gly:AA molar ratio. This clearly shows that problematic end-of-life tires can be successfully valorized into efficient carbon-type catalysts with promising activities in acetin production, contributing to the recycling of troublesome rubber wastes.

## 3. Materials and Methods

Two recovered carbon blacks (rCB) obtained from used tires and supplied by Contec (Poland) were functionalized with sulfonic groups and used as catalysts for glycerol acetylation to produce acetins. A commercial carbon black (CB, purchased from Cabot Corporation) sample was employed as a reference material. 

### 3.1. Recovered Carbon Black Preparation

The pristine rCBs (labeled as rCB_1 and rCB_2) were produced by pyrolyzing waste passenger tires using two different proprietary technologies [80]. Some details of the applied production methods are presented below. 

The rCB_1 sample was acquired via periodic (batch) pyrolysis conducted at a temperature of 450–550 °C in a rotary kiln. In this process, rubber granules made from shredded tires were utilized and placed inside the reactor. The kiln was heated from the bottom, and mixing was achieved through the rotary motion of the pyrolysis chamber.

In the second method, continuous pyrolysis of whole tires occurred in an Auger-type reactor with a rotating shaft at a temperature of 510 °C and using molten salt as the heating medium (the so-called Molten technology). The final sample obtained in this method was denoted as rCB_2. 

In laboratory conditions, the steel wires and cords from the post-pyrolytic solids were removed by sieving. The carbon particles of sizes ≤ 0.4 mm were collected.

### 3.2. Functionalization of the Samples

To endow the samples with acidic properties, the obtained rCBs as well as a commercial carbon black (CB) were modified with concentrated sulfuric acid (96 wt.%). The process was performed in a three-neck bottom flask equipped with a condenser under Ar flow and continuous stirring. In each case, 3.5 g of sample and 90 mL of H_2_SO_4_ (abbreviated here ‘SA’) were mixed and heated up to 140 °C. The reaction was performed for 20 h, after which the reaction mixture was cooled down, diluted with distilled water, and filtrated. The collected sample was further washed thoroughly with hot distilled water until the filtrate pH was neutral. Finally, the material was dried at 110 °C overnight and sieved to a uniform particle size ≤ 0.4 mm. The samples were labeled according to the following scheme: type of carbon sample (i.e., rCB_1, rCB_2, or CB)-SA, where SA = concentrated sulfuric acid.

### 3.3. Characterization of the Samples

The elemental C, H, N, S compositions of the samples were analyzed using an Elemental Analyzer Vario El III. The ash contents were determined as residue after combustion at 815 °C for 2 h. The total acidity of the materials was measured by a potentiometric back titration method using a Cerko Lab microtitrator unit. For this purpose, ~100 mg of the sample was mixed with 50 mL of a 0.01 M NaOH solution and shaken at ambient temperature for 20 h. Afterward, the suspensions were filtrated and the filtrate was titrated with a 0.05 M HCl solution. A blank test (without a sample) was also performed, and the obtained value was taken for the calculation. The crystallographic structure of both post-pyrolytic and sulfonated recovered carbon blacks (rCBs) was examined via X-ray diffraction (XRD) analysis, performed with a Bruker D8 Advance diffractometer and using Cu Kα radiation (λ = 1.54056Å). The XRD scans were carried out within the range of 2θ from 6° to 60°. The textural characteristics of the studied materials were evaluated via nitrogen (N_2_) adsorption/desorption measurements at a temperature of −196 °C, employing a Quantachrome Nova 1000e sorptometer, Boynton Beach, FL, USA. Before these measurements, the samples underwent a vacuum outgassing process at 90 °C. The specific surface area was determined using the BET equation. The micropore volumes (*V_micro_*) were calculated by the t-plot method. In turn, the total volumes of pores (*V_tot_*) were measured from the amount of N_2_ adsorbed at a relative pressure close to 1. X-ray photoelectron spectroscopy (XPS) measurements were conducted using an Ultra High Vacuum (UHV) System from Specs, Berlin, Germany. The materials under examination were exposed to monochromatic Al Kα radiation (1486.6 eV). The chamber operated at an approximate pressure of 2 × 10^−9^ mbar. Binding energies (BE) were calibrated against the C1s peak originating from the carbon surface layer, set at 284.5 eV. Spectroscopic data were processed through CasaXPS software (version 2.3.25PR1.0) developed by Casa Software Ltd., Teignmouth, UK, employing a peak-fitting algorithm along with a Shirley background correction. A microscopic analysis was conducted on the powder to investigate the morphology of the particles. A small quantity of the uncoated powder pellets was placed on a metallic slide and examined using a scanning electron microscope (SEM, Hitachi SU3500, Tokyo, Japan). TEM analysis was conducted using the FEI Tecnai G^2^ 20 X-Twin Electron Microscope operating at a voltage of 200 kV. Energy-dispersive X-ray spectroscopy (EDX) was integrated with transmission electron microscopy (TEM) to identify various elements within the samples. TG measurements were performed using a Setsys 1200 Setaram analyzer (Caluire, France) by heating the samples to 1000 °C in an N_2_ flow (with a temperature rise of 10 °C/min).

### 3.4. Catalytic Tests

The tire-derived carbons were tested as catalysts for glycerol esterification. The process was performed under solventless conditions and atmospheric pressure in an Ar flow using acetic acid (AA). AA (28.9 mL) was mixed with 0.7 g of a previously dried catalyst and heated to 110 °C. After the desired temperature was reached, glycerol (Gly; 7.75 g; Gly:AA molar ratio of 1:6) was added to the flask, and this time point was treated as t = 0 h. To monitor the process, aliquots of the reaction mixtures were withdrawn after different time intervals (1, 2, 4, 6, and 24 h), and the catalyst was separated from the liquid mixture by centrifugation. After dilution of the supernatant fluid with ethanol, the composition of the reaction mixture was analyzed employing an SRI 8610C gas chromatograph equipped with a flame ionization detector (FID) and an InertCap WAX capillary column working at a temperature range of 130–210 °C under Ar flow. After finishing the esterification process, the catalyst was recovered by filtration, washed with hot distilled water followed by washing with acetone, and dried overnight at 110 °C. Afterward, it was used in a subsequent reaction cycle. To confirm the catalytic properties of the prepared samples, a blank test (without a catalyst) was also performed. For the sake of comparison, modified commercial carbon black (CB-SA) was also tested. The catalytic performances of the samples were expressed as the conversion of glycerol (*X_Gly_*), selectivities to various acetins (*S_MA_*, *S_DA_*, and *S_TA_*), and/or yields of different products (*Y_MA_*, *Y_DA_*, and *Y_TA_*).

## 4. Conclusions

rCB produced from post-consumer tires were shown to be transformable to efficient catalysts of the acetins production through glycerol acetylation. Tuning the surface properties and introducing specific functional groups on the rCBs were achieved by functionalization of the samples with concentrated sulfuric acid. This modification was proved to effectively introduce -SO_3_H moieties onto the surface of rCBs and increase the total acidity of the treated materials. Partial removal of inorganic substances from the sample structure was also observed. 

The functionalized rCBs were active in glycerol acetylation, giving a very high conversion of glycerol (85–90%) within 1 h. Furthermore, the samples showed significant activity towards the formation of diacetins and triacetin, yielding 70–75% of DA + TA within 4 or 6 h. The catalytic effects presented by the modified rCBs were induced by the sulfonic groups present on the surface of the samples and were comparable with those achieved with the sulfonated commercial CB. The functionalized rCBs remained catalytically stable in subsequent reaction runs, with only slight changes in glycerol conversion and DA and TA yields. 

## Figures and Tables

**Table 1:** The results of the elemental analysis (in wt.%), ash determination (wt.%), and total acidity measurements (A_tot_, mmol H^+^/g) for the pristine and modified rCB and CB samples.

Sample	Ash	C	H	N	S	O *	A_tot_
CB	0.0	99.6	0.1	0.0	0.0	0.2	0.03
CB-SA	0.0	97.4	0.1	0.0	1.0	0.8	0.30
rCB_1	20.7	79.6	0.6	0.4	2.8	ns ^#^	0.04
rCB_1-SA	11.3	76.1	0.5	0.4	3.8	7.9	0.61
rCB_2	26.8	74.7	0.9	0.3	2.4	ns ^#^	0.09
rCB_2-SA	12.6	73.5	0.4	0.3	3.7	9.5	0.76

* calculated by the difference; # ns, not specified due to the negative value of the calculation (possible as the ash yields ≠ the mineral matter [54]).

**Table 2:** Textural analysis of the commercial and post-pyrolytic carbon blacks (CB and rCBs, respectively) and their modified counterparts.

Sample	*S_BET_* (m^2^/g)	V_tot_ (cm^3^/g)	D (nm)
CB	68	0.40	23.5
CB-SA	78	0.55	27.9
rCB_1	66	0.30	22.4
rCB_1-SA	75	0.35	19.3
rCB_2	63	0.40	31.1
rCB_2-SA	81	0.38	25.9

**Table 3:** The results of the elemental analysis and ash determination (in wt.%) as well as total acidity measurements (A_tot_, in mmol H^+^/g) for the rCB_1-SA sample after four acetylation runs.

Sample	Ash	C	H	N	S	O *	A_tot_
rCB_1-SA after 4th cycle	11.7	78.6	0.8	0.7	2.2	6.0	0.52

* calculated from the difference.

**Table 4:** Comparison of the catalytic performance of the obtained catalysts with activities of other reported carbon systems.

Catalyst	Operating Conditions	Catalytic Performance	Ref.
C_glycerol	110 °C, Gly:AA molar ratio = 1:6, *t* = 2 h	*X_Gly_* = 97%, *S_DA_* = 56%, *S_TA_* = 23%	[25]
SHTC	115 °C, Gly:AA molar ratio = 1:9, *t* = 4 h	*X_Gly_* ≈ 100%, *S_DA_* ≈ 60%, *S_TA_* ≈ 30%	[76]
TRGO_BDS	110 °C, Gly:AA molar ratio = 1:6, *t* = 2 h	*X_Gly_* = 97%, *S_DA_* = 59%, *S_TA_* = 20%	[77]
AC_micro__BDS	110 °C, Gly:AA molar ratio = 1:6, *t* = 4 h	*X_Gly_* = 97.5%, *S_DA_* = 55%, *S_TA_* = 22.5%	[35]
AC-SA5	120 °C, Gly:AA molar ratio = 1:8, *t* = 3 h, pressurized reactor	*X_Gly_* = 91%, *S_DA_* = 28%, *S_TA_* = 34%	[78]
KJ-400	120 °C, Gly:AA molar ratio = 1:5, *t* = 4 h	*X_Gly_* = 88.5%, *S_DA_* = 40%, *S_TA_* = 4%	[79]
C-SA	120 °C, Gly:AA molar ratio = 1:5, *t* = 2 h	*X_Gly_* = 98.4%, *S_DA_* ≈ 54.5%, *S_TA_* ≈ 13%	[47]
rCB_2-SA	110 °C, Gly:AA molar ratio = 1:6, *t* = 4 h	*X_Gly_* = 95%, *Y_DA_* = 55% (*S_DA_* = 58%) *Y_TA_* = 16.5% (*S_TA_* = 17.4%)	This work

Gly—glycerol; AA—acetic acid; C_glycerol—carbon obtained from glycerol by partial carbonization with sulfuric acid; SHTC—hydrothermal carbon from glucose sulfonated with sulfuric acid; TRGO_BDS—thermally reduced graphene modified with diazonium salt; AC_micro_-BDS—microporous activated carbon modified with diazonium salt; AC-SA5—commercial activated carbon modified with 5 M sulfuric acid; KJ-400—carbon obtained from Karanja seed shell at 400 °C; C-SA—sulfonated carbon from willow catkins; rCB_2-SA—recovered carbon black from tires obtained using Molten technology and sulfonated with sulfuric acid.

## Data Availability

The data presented in this study are available on request from the corresponding author. The data are not publicly available due to partner industry agreements that require proprietary information to be kept confidential.

## Supplementary Materials

The following supporting information can be downloaded at: https://www.mdpi.com/article/10.3390/molecules28248137/s1; Figure S1: SEM images of a commercial CB in lower (a) and higher (b) magnification; Figure S2: The EDX spectra of pyrolytic carbon black samples taken from different spots: (a) and (b) rCB_1; (c) and (d) rCB_2; Figure S3: The high-resolution XPS S 2p spectra of rCB_2 (a), and rCB_2-SA (b); Figure S4: The high-resolution XPS C 1s spectra of (a) rCB_1, (b) rCB_1 modified with sulfuric acid, (c) rCB_2, and (d) rCB_2 modified with sulfuric acid; Figure S5: The N_2_ adsorption-desorption isotherms of the pristine and sulfonated rCB_1 (a) and rCB_2 (b).
