# Supplementary material for: Upgrading Pyrolytic Residue from End-of-Life Tires to Efficient Heterogeneous Catalysts for the Conversion of Glycerol to Acetins
Source: Molecules. 2023 Dec 17;28(24):8137. doi: 10.3390/molecules28248137 (PMC10745658; doi:10.3390/molecules28248137)
Supplement: Supplementary file 1 [file molecules-28-08137-s001.zip › molecules-2718988-supplementary.pdf]

SUPPLEMENTARY MATERIAL

Upgrading Pyrolytic Residue from End-of-Life Tires to Efficient Heterogeneous Catalysts for the Conversion of Glycerol to Acetins

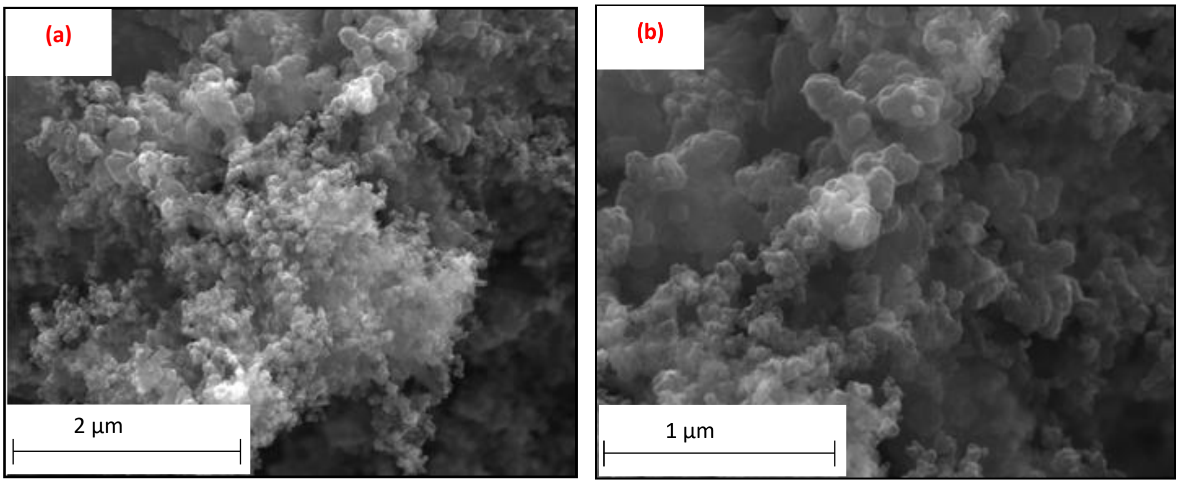

Figure S1. SEM images of a commercial CB in lower (a) and higher (b) magnification (the sample analyzed using FEI Quanta 400 FEG ESEM/EDAX Pegasus X4M apparatus).

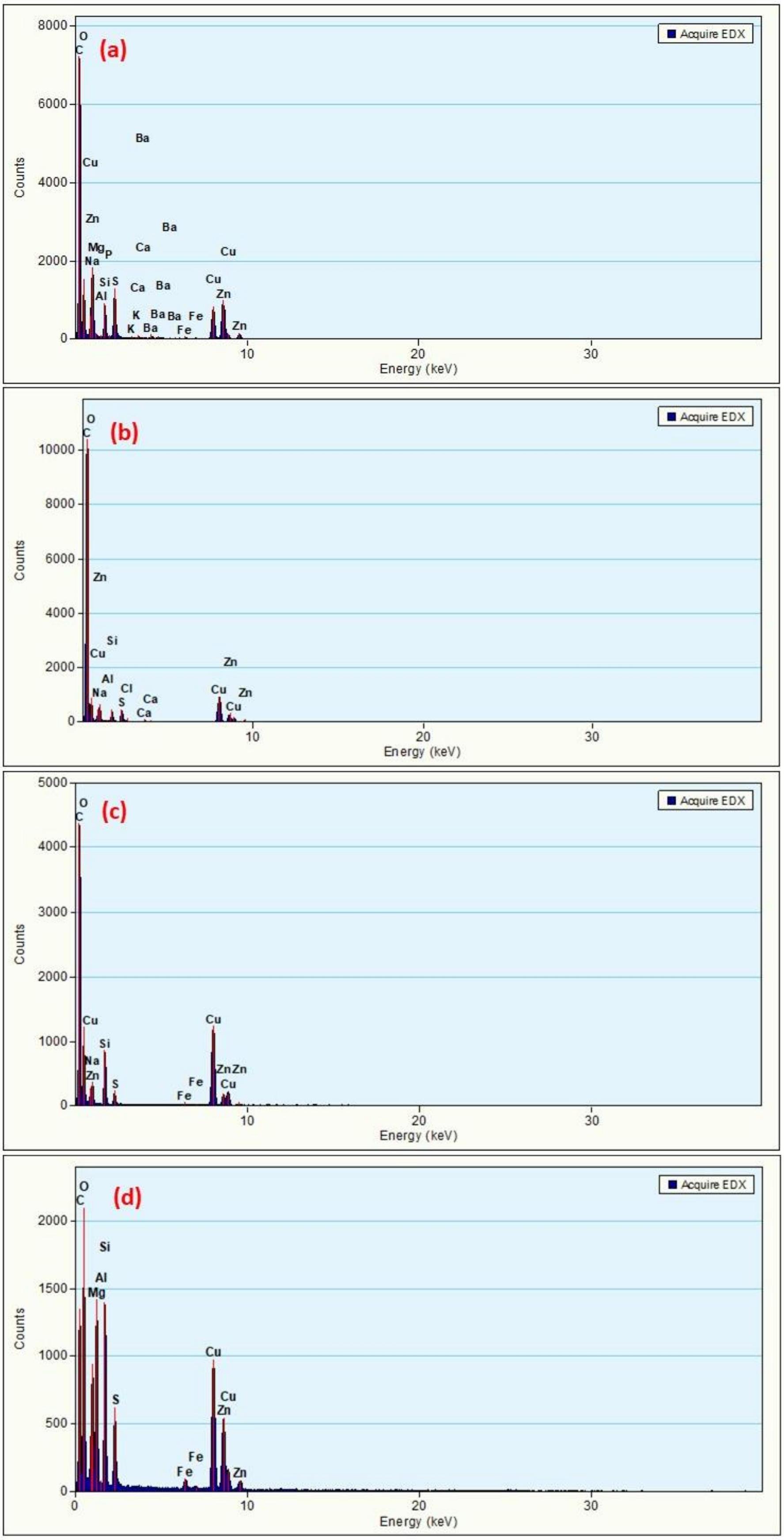

Figure S2. The EDX spectra of pyrolytic carbon black samples taken from different spots: (a) and (b) rCB_1; (c) and (d) rCB_2.

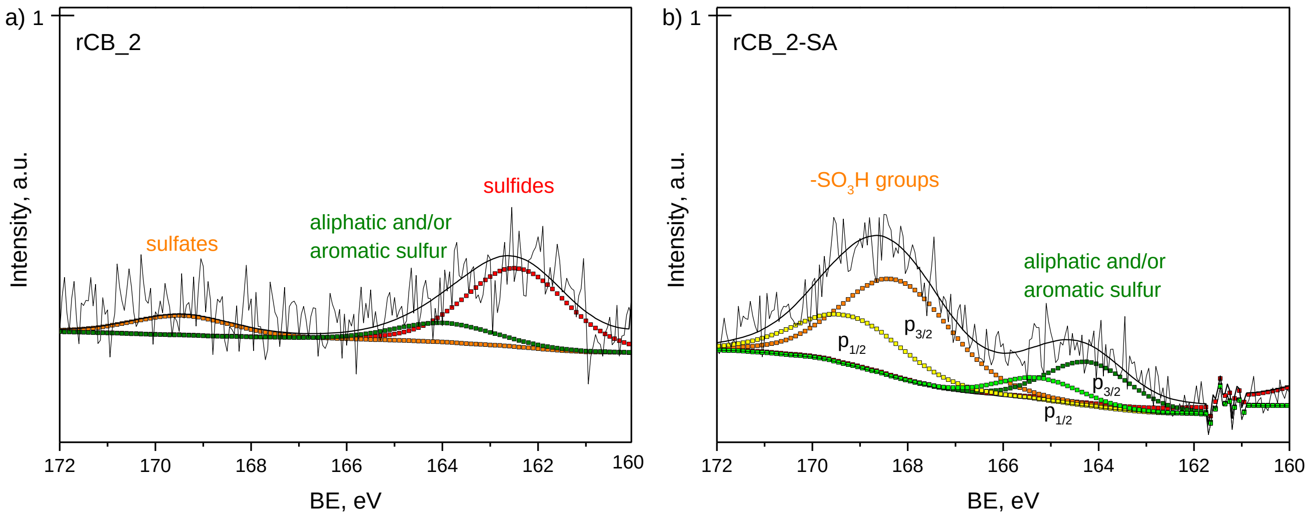

Figure S3. The high-resolution XPS S 2p spectra of rCB_2 (a), and rCB_2-SA (b) (spin-orbit splitting in (a) omitted for clarity).

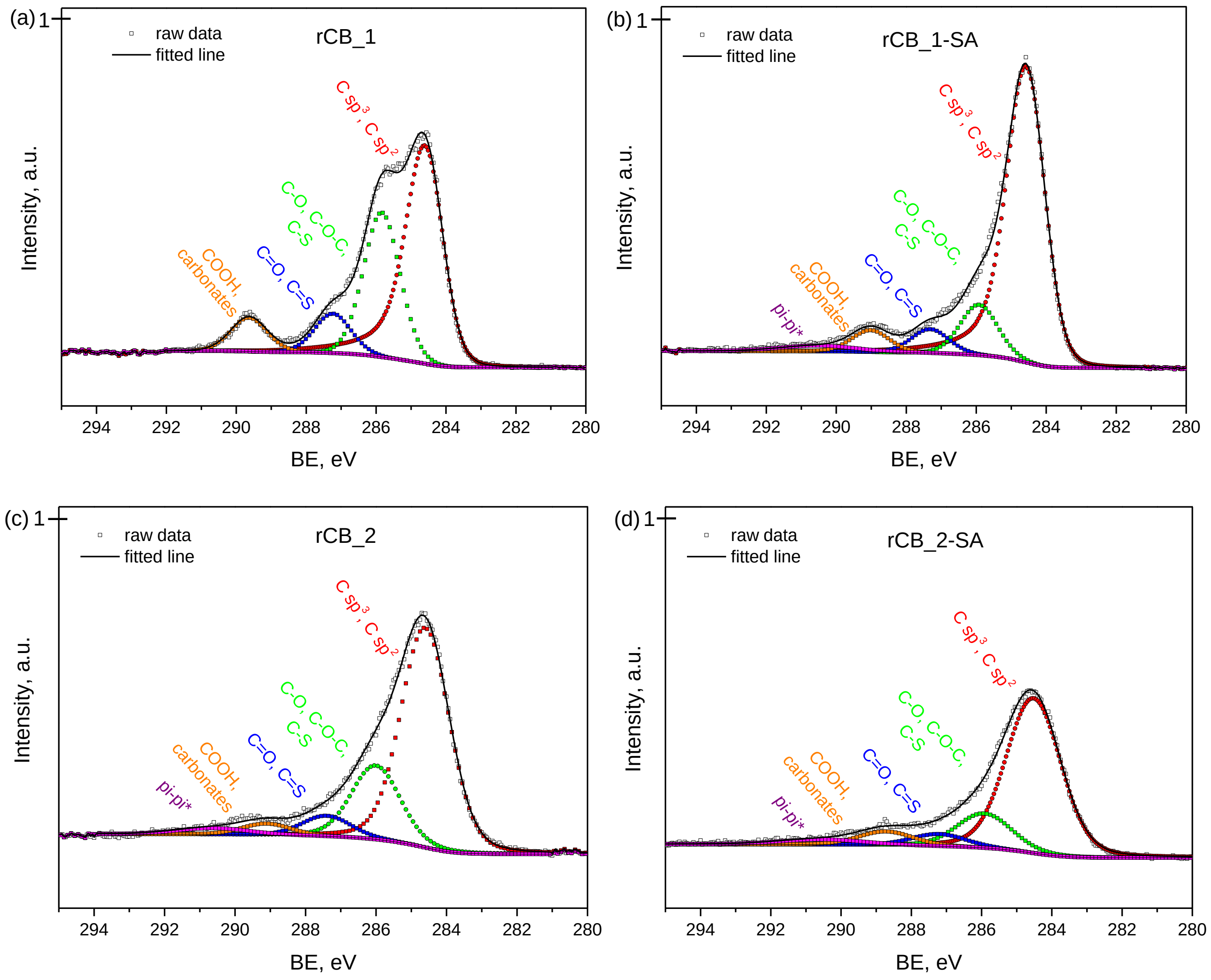

Figure S4. The high-resolution XPS C 1s spectra of (a) rCB_1, (b) rCB_1 modified with sulfuric acid, (c) rCB_2, and (d) rCB_2 modified with sulfuric acid.

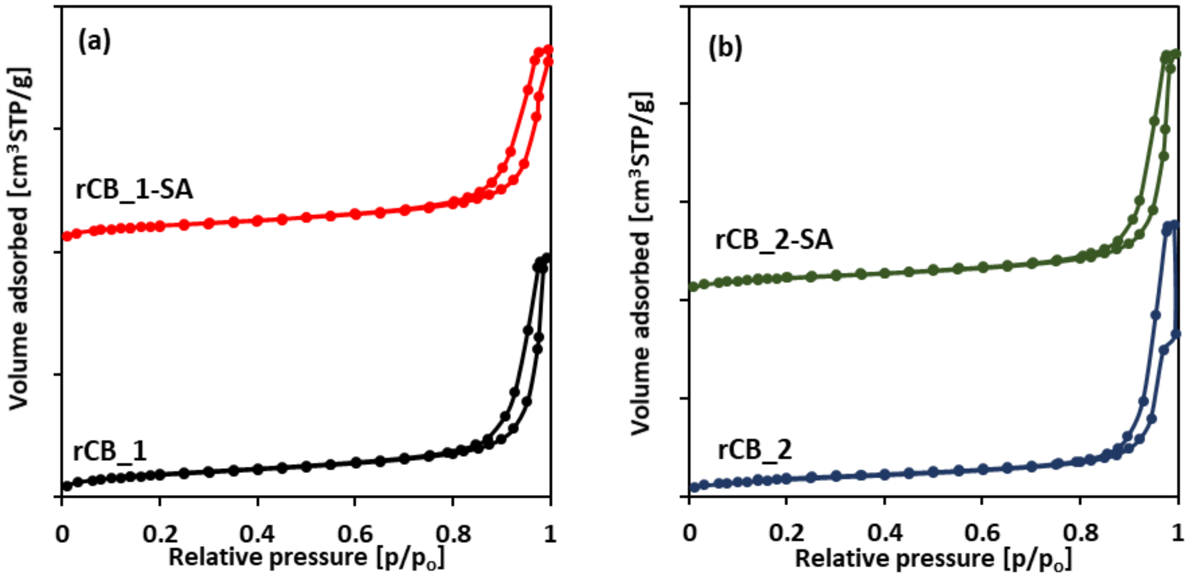

Figure S5. The N_2 adsorption-desorption isotherms of the pristine and sulfonated rCB_1 (a) and rCB_2 (b).
